# Video Q&A: Non-coding RNAs and eukaryotic evolution - a personal view

**DOI:** 10.1186/1741-7007-8-67

**Published:** 2010-07-16

**Authors:** John Mattick

**Affiliations:** 1 Institute for Molecular Bioscience, Queensland Bioscience Precinct, 306 Carmody Road, The University of Queensland, St Lucia, QLD 4072, Australia

## John Mattick talks on the modern world of non-coding RNA

## 

John Mattick graduated from the University of Sydney in 1972 and finished his PhD from Monash University in 1977, after which he entered on postdoctoral studies on fatty acid synthase at Baylor College of Medicine in Houston. While in Houston he first became interested in the question of whether non-coding RNA has a function, when introns were discovered in the coding sequences of genes. But most of his work for the next 25 years was in microbiology, and it was not until the genomic studies of the past 15 years, and the revelation that most of the non-coding DNA of the human genome is transcribed, that he turned in earnest to the question of what the non-coding transcripts might be contributing. This is now the focus of his laboratory at the Institute for Molecular Biosciences at the University of Queensland, where he has worked since 1988.

In this interview, he explains why he thinks non-coding RNA is fundamental to eukaryotic evolution.

## Edited transcript

### When people talk about the RNA world, they usually mean a pre-protein world, but you would say there is a largely unexplored RNA world today. Why?

The thesis that RNA was the primordial molecule of life is compelling because RNA has both functional and information-carrying capacity. But there's no reason to think those capacities were ever lost. It does appear that early in the evolution of cellular life RNA devolved its informational storage functions to DNA, as a much more stable and easily replicable molecule, and its analog functions to proteins, which have much greater chemical versatility. So on that basis the idea grew up that RNA had become an ephemeral intermediate between the hard disk - the DNA - and the analog outputs, the proteins. But what I think then happened is that later in evolution RNA re-entered the scene to fulfill a regulatory imperative associated with the emergence of developmentally complex organisms, acquiring a whole range of functions based on those same primordial properties of sequence specificity and the ability to fold into complex shapes to interact with other molecules in specific and dynamic ways.

### But we know that proteins have regulatory functions, and can interact in many ways. Why postulate regulatory RNA?

There are a few key points. The first - and this is one of the great surprises of the genome projects, that very few people have commented on because of their background assumptions - is that both the number and range of protein-coding genes have remained largely the same since the base of the metazoan radiation. *Caenorhabditis elegans*, which is a worm of only 1,000 cells, has almost precisely the same number of protein-coding genes as a human - about 20,000 is the latest estimate - and most of those genes encode similar functions. So the basic parts set for animal development was established several hundred million years ago. In fact, I understand the sponge genome also encodes most, if not all, of the key protein families that are involved in regulating development. Now *C. elegans *has only got 1,000 cells - a few muscle cells, a few nerve cells, and a gut. We humans have 30 trillion to 100 trillion cells, and the complexity of our body plan organization - including all of the muscles in the face that reflect the range of human emotions, the different bones and organs, and the brain - is enormous.

### So did the limited diversity of proteins in phylogeny lead to the suggestion that non-coding RNA might have important regulatory functions?

Yes. Since the protein-coding repertoire (notwithstanding some clade-specific innovations) has remained relatively static, the differences in developmental complexity must be due to an expansion of the accompanying regulatory architecture, which presumably lies outside the protein-coding sequences. Now, interestingly, that problem, I think, has been swept under the intellectual carpet because of the relatively facile and widely accepted assumption, which has not been challenged, nor justified, that the combinatorics of transcription factors provide an explosive number of regulatory possibilities - with enough capacity in the system to program anything from a worm to human. But you certainly need to have a more complex regulatory framework to get to a more complex organism, and the astounding thing is that the only thing that does scale with complexity - because the number of genes does not - is the extent of the non-protein-coding genome.

Now of course that's going to include regulatory elements, but it's so large - in humans 98.8% - that most molecular biologists have not considered that this could all be regulatory and have consequently assumed that most of it must be just evolutionary debris - a view that was compounded by the fact that roughly half our genome derives from transposons - something we might come back to.

In any case, protein-coding genes do not scale with complexity, whereas the non-coding genome does, at least to first approximation. And here's the interesting thing: surprisingly, virtually all of these non-coding sequences are transcribed into non-protein-coding RNAs, apparently in a differential fashion that seems to be developmental-stage specific, tissue specific, and cell specific. So there are only two alternatives, which is what occurred to me back in 1978 when I first bumped into introns as a postdoctoral fellow. At the time it was universally assumed - by everybody, including Crick - that because these sequences did not code for protein they must be junk, and they were rationalized as hangovers of early evolution. At the time I remember thinking to myself that this was a very strange observation. Huge genes are transcribed into RNA and then the RNA introns are cut out and apparently discarded. So, yes, one possibility is that the RNA is junk and this is just useless recycling of ribonucleotides. But the other possibility is, and was then, that the expressed non-coding RNA is functional. This to me was much more interesting, indeed exciting, with potentially profound consequences. So it became my intellectual hobby to explore the idea, although in those days there were very few tools with which to do so - so for a long time it simmered on my backburner while I did more conventional things.

### But doesn't the relative non-conservation of non-coding RNA mean that it can't have important functions?

The level of conservation is an old chestnut, and in your question about the relative conservation is in fact embedded the answer. The non-coding RNAs that are differentially transcribed and developmentally regulated are on the whole less conserved than protein-coding sequences. But lack of relative conservation does not mean lack of function. Conservation is imposed by structure-function relationships, which vary between different types of sequences. Structure-function relationships in most proteins are very strict. There are only so many ways to make an oxygen-binding protein, or a wheel for that matter. Analog functions have particular structural imperatives. But regulatory sequences can be much more plastic, just like your credit card. It doesn't mean they don't have important information and indeed I think most people - even those who are sceptical about the level of importance of RNAs - would acknowledge that most phenotypic radiation occurs in the regulatory architecture. We take a relatively common set of components and arrange their expression in different ways to produce a range of phenotypic outcomes both between species and within species.

### Are you arguing that you wouldn't expect regulatory RNAs to be conserved?

There is not a lack of conservation of regulatory RNAs. Indeed some are very highly conserved. In general, however, they have a lower relative conservation compared with sequences encoding proteins. The level of conservation of regulatory sequences varies, reflecting the greater plasticity of regulatory molecules and the fact that this is where evolution is selecting, initially positively, and subsequently negatively, for regulatory variation that underpins phenotypic radiation.

### So do you believe that we simply haven't understood the regulatory mechanisms underlying evolution?

It does seem that we've fundamentally misunderstood the structure of genetic programming of higher organisms because of the assumption, which is largely true for bacteria, but turning out not to be true for the complex eukaryotes, that most genetic information is transacted by proteins. The evidence, dating back in fact to 1977, is that there is a vast hidden layer of regulatory RNAs that are involved in directing the epigenetic trajectories of differentiation and development, and this is now just beginning to be peeled back.

### What is the evidence for regulatory functions for non-coding RNAs?

Perhaps the best way to answer the question is to give two examples of how these RNAs are functioning and why the system has superimposed an RNA regulatory system on top of a protein-based regulatory system. The first is microRNAs, which were discovered ten years ago through some terrific genetics in *C. elegans *in the preceding decade. MicroRNAs are now known to regulate virtually all known developmental processes in animals and plants. They have no known catalytic function - they are just 22 or so nucleotides that target another RNA, and the resulting complex, in some fashion that's not fully understood, is then recognized and acted upon by a generic protein complex, the so-called RISC complex. The cell, and indeed evolution, can dial up these microRNAs very flexibly in different cells to address various targets, and they only need one protein complex to come and do the job. So the signal has been separated from the consequent analog action, and instead of having one protein or protein complex for every regulatory event, its function has been allocated to a single generic complex which is directed to different targets using much more genomically compact and evolutionarily flexible small RNAs.

### That's one example of a regulatory function. What's the other?

It's not as well accepted yet, but it is looking increasingly likely that an analogous process occurs in the regulation of chromatin modification and epigenetic processes. The modulation of chromatin structure and epigenetic memory is critical to development of complex organisms. Chromatin architecture is controlled by DNA methylases and a set of relatively generic enzymes and enzyme complexes that modify histones in different ways: about 60 of them in all. What determines their selectivity, at myriad different sites around the genome, is not known, but it had been assumed to be 'transcription factors' - itself a very vague term. However it's looking increasingly as though the site selectivity of these enzymes is actually being controlled by RNAs that provide the sequence-specific signals with the adaptor functions that then recruit generic protein complexes at the relevant sites of action during differentiation and development. And now there's good evidence from our lab and others that at least a subset of the long non-coding RNAs that are differentially expressed during development fulfill this function, because they associate physically with complexes involved in chromatin modification.

### Are there any specific examples of regulatory functions of non-coding RNAs in development?

We've pinned function to a few. There are tens if not hundreds of thousands of long non-coding RNAs. Very few have been studied in detail: I recently wrote a review for *PLoS Genetics *that lists those for which there are good functional data, of which there are about 40 or so. That's a small number, but it's enough to give you an idea. For example, we and others have shown that one of these non-coding RNAs is required for the formation of paraspeckles, a sub-nuclear compartment that's induced upon cellular differentiation. Other non-coding RNAs are associated with chromatin complexes; and some non-coding RNAs have been shown by biological assays to be critical for such things as eye development, and some have been associated with different sorts of diseases, including heart disease and cancer.

### So there's not very much direct functional evidence yet?

It's early days. In fact almost every time you functionally test a non-coding RNA that looks interesting because it's differentially expressed in one system or another, you get functionally indicative data coming out. But the compelling point is that regulatory RNAs provide an explanation as to why complexity doesn't scale with the number of protein-coding genes. It was originally assumed that as complexity increased there would be more and more such genes - before the genome was sequenced there was speculation that humans might have a hundred thousand or more, and it was a huge shock that it's much less, and doesn't scale with complexity. But there are very large numbers of long non-coding RNAs, so this is where the real genetic scaling has occurred.

### You mentioned that non-coding RNAs are implicated in disease. Could they explain why in genome-wide association studies disease-associated polymorphisms turn up in non-coding regions of the genome?

It's perfectly possible. There's no doubt that in genome-wide association studies looking into the genetic components of complex diseases and complex traits, most of the mapped locations are non-coding and therefore almost by definition regulatory. So it's really a question of what form that regulatory variation takes. But there's an important point here. In the early days of human gene mapping, people were searching for the genes responsible for diseases such as cystic fibrosis, Huntington's disease, thalassemias and so on, which cause what I call catastrophic component damage: if you lack a functional protein component, it's like losing a light switch or a wheel - in most cases it's a very serious problem. So the genetic signature is very strong, and the gene is relatively easy to map. But with complex diseases, there are often multiple genetic components, which are very difficult to map. It turns out that most of the classic monogenic diseases are caused by protein-coding mutations. However, not surprisingly, most of the genetic variation that affects complex human traits appears to lie in regulatory mutations. Well over 90% of all the loci mapped in genome-wide association studies are non-coding, and many of them are miles from any coding sequences. It is possible that all of these could be conventional *cis*-acting promoter or enhancer mutations affecting DNA sequences recognized by regulatory proteins - but intriguingly, at least some of these loci are turning out to be in non-coding regions that are differentially expressing non-coding RNAs.

Indeed, I'd like to emphasize the following point about the expression of non-coding RNAs: it is extraordinarily specific, both spatially and temporally. For example, we did a study in conjunction with the Allen Institute for Brain Science in Seattle in which we looked at well over 1,000 of these non-coding RNAs, and found that half are expressed in brain and show extremely precise spatial expression. Some are only expressed in the dentate gyrus of the hippocampus, others in particular layers of the cortex, and others in Purkinje cells in the cerebellum. Moreover, in 80% of the cases where we had sufficient resolution to tell, these RNAs are trafficked to specific subcellular locations. So this is not some fuzzy random signal: their expression is extremely precise, both in terms of the cell specificity and in terms of subcellular localization. That seems to me to have none of the characteristics you would expect if these RNAs are just some sort of background noise. On the contrary, I think the differential expression of these RNAs is the only reliable genome-wide index of their function.

### You mentioned earlier the possible significance of transposons. What part do you think they have played?

That is one of my many favourite topics. It is widely assumed - though not by everybody - that transposon-derived sequences are simply 'selfish' mobile genetic elements that have no function other than their own propagation. Books have been written about such things, and that is indeed one possibility. But the raw material for evolution is duplication and transposition, with the latter having the great advantage of being able to distribute functional cassettes. So it's equally possible that a large fraction of the transposon-derived sequences that are in our genome are actually functional.

### It's not generally believed that transposon sequences have regulatory functions, is it?

I predict that there will be a very rapid change of attitude to transposon-derived sequences. We are already seeing papers showing their differential expression. Many of them are transcribed by RNA polymerase III, so they have been under the radar of poly(A)-based approaches to the transcriptome. But I predict we are going to see that they are critical drivers of evolution - critical in embryogenesis and development, and extremely critical in the brain.

### Is there anything you can say to support the prediction that regulatory RNA will be particularly important in the brain?

One point about RNA that has really not penetrated the consciousness of most biologists yet is that it is extensively edited, and by editing I mean deamination of adenosines to form inosines, and cytosines to form uracil, which changes the sequence and structure of the RNA. RNA-editing enzymes have expanded greatly during vertebrate, mammalian and primate evolution. They occur in most, if not all, tissues, but are especially active in the brain. Some are brain specific, and RNA editing is approximately 30 times more intensive in the human brain than in the mouse. So it seems to me increasingly obvious that RNA editing is the principal means by which environmental information is transmitted to the epigenome, and is the mechanism for connecting the environment to the genome, the expansion of which was critically important to the evolution of the plasticity and the molecular mechanisms of learning and memory. In other words, RNA regulation is central not only to development, but also to the ability to plastically alter the genetically encoded information without changing the hard-wired DNA (although that may occur in some cells as well). That makes it the key to the evolution of cognition.

### Where can I find out more?

Articles

Mattick JS: **RNA regulation: a new genetics? ***Nat Rev Gene *2004, **5:**316-323.

Pang KC, Frith MC, Mattick JS: **Rapid evolution of noncoding RNAs: lack of conservation does not mean lack of function. ***Trends Genet  *2006, **22:**1-5.

Taft RJ, Pheasant M, Mattick JS: **The relationship between non-protein-coding DNA and eukaryotic complexity. ***BioEssays*2007, **29:**288-99.

Mattick JS: **A new paradigm for developmental biology. ***J Exp Biol *2007, **210:**1526-47.

Amaral PP, Dinger ME, Mercer TR, Mattick JS: **The eukaryotic genome as an RNA machine. ***Science *2008, **319:**1787-1789.

Amaral PP, Mattick JS: **Noncoding RNA in development. ***Mamm Genome  *2008, **19:**454-492.

Dinger ME, Amaral PP, Mercer TR, Pang KC, Bruce SJ, Gardiner BB, Askarian-Amiri ME, Ru K, Soldà G, Simons C, Sunkin SM, Crowe ML, Grimmond SM, Perkins AC, Mattick JS: **Long noncoding RNAs in mouse embryonic stem cell pluripotency and differentiation. ***Genome Res   *2008, **18:**1433–1445.

Mercer TR, Dinger ME, Sunkin SM, Mehler MF, Mattick JS: **Specific expression of non-coding RNAs in mouse brain. ***Proc Natl Acad Sci USA  *2008, **105:**716-721.

Mattick JS, Mehler MF: **RNA editing, DNA recoding and the evolution of human cognition. ***Trends Neurosci   *2008, **31:**227-233.

Mattick JS, Amaral PP, Dinger ME, Mercer TR, Mehler MF: **RNA regulation of epigenetic processes. ***BioEssays  *2009, **31:**51-59.

Guttman M, Amit I, Garber M, French C, Lin MF, Feldser D, Huarte M, Zuk O, Carey BW, Cassady JP, Cabili MN, Jaenisch R, Mikkelsen TS, Jacks T, Hacohen N, Bernstein BE, Kellis M, Regev A, Rinn JL, Lander ES: **Chromatin signature reveals over a thousand highly conserved large non-coding RNAs in mammals. ***Nature  *2009, **458:**223-227.

Mattick JS: **The genetic signatures of noncoding RNAs. ***PLoS Genet   *2009, **5:**e1000459.

Khalil AM, Guttman M, Huarte M, Garber M, Raj A, Rivea Morales D, Thomas K, Presser A, Bernstein BE, van Oudenaarden A, Regev A, Lander ES, Rinn JL: **Many human large intergenic noncoding RNAs associate with chromatin-modifying complexes and affect gene expression. ***Proc Natl Acad Sci USA   *2009, **106:**11667-11672.

Taft RJ, Glazov EA, Cloonan N, Simons C, Stephen S, Faulkner GJ, Lassmann T, Forrest ARR, Grimmond SM, Schroder K, Irvine K, Hume DA, Suzuki H, Orlando V, Carninci P, Arakawa T, Nakamura M, Kubosaki A, Hayashida K, Kawazu C, Murata M, Nishiyori H, Fukuda S, Kawai J, Daub CO, Hayashizaki Y, Mattick JS: **Tiny RNAs associated with transcription start sites in animals. ***Nat Genet   *2009, **41:**572-578.

Taft RJ, Pang KC, Mercer TR, Dinger ME and Mattick JS: **Noncoding RNAs: regulators of disease. ***J Pathol    *2010, **220:**126-139.

Mattick JS, Taft RJ, Faulkner GJ: **A global view of genomic information - moving beyond the gene and the master regulator. ***Trends Genet   *2010, **26:**21-28.

## Supplementary Material

John Mattick talks on the modern world of non-coding RNA Click here for file

